# Postoperative Management Strategy of Surgical Site Infection following Lumbar Dynesys Dynamic Internal Fixation

**DOI:** 10.1155/2021/2262837

**Published:** 2021-10-07

**Authors:** Liehua Liu, Lei Luo, Chen Zhao, Qiang Zhou

**Affiliations:** ^1^Department of Spine Surgery, The Third Affiliated Hospital of Chongqing Medical University, Chongqing, China; ^2^Department of Orthopedics, Southwest Hospital, Army (Third) Military Medical University, Chongqing, China

## Abstract

**Aim:**

To research the incidence of surgical site infection (SSI) following lumbar Dynesys dynamic internal fixation and its management strategy.

**Methods:**

We retrospectively analyzed all cases of lumbar Dynesys dynamic internal fixation performed from January 2010 to December 2019, and the data from patients with SSI were collected. The observational indicators included the incidence of SSI, general information of the patients, surgical details, inflammatory indicators, pathogenic bacteria, and treatment. SSI was defined as both early infection and delayed infection, and the cases were divided into Groups A and B, respectively. The relevant indicators and treatment were compared between the two groups.

**Results:**

A total of 1125 cases of lumbar Dynesys dynamic internal fixation were followed up. Twenty-five cases of SSI occurred, and the incidence of SSI was 2.22% (25/1125). There were 14 cases of early infection (1.24%) and 11 cases of delayed infection (0.98%). Fourteen cases of early infection occurred 12.3 ± 8.3 days postoperatively (3–30), and 11 cases of delayed infection occurred 33.3 ± 18.9 months postoperatively (3–62). The inflammatory indicators of Group A were significantly higher than those of Group B (all *P* < 0.05), except for procalcitonin. The main infection site in Group A was located on the skin and subcutaneous tissue and around the internal instrument, while the main infection site in Group B was around the internal instrument. The main treatment for Group A was debridement and implant replacement, and the main treatment for Group B was implant removal. *Summary*. The incidence of SSI following lumbar Dynesys dynamic internal fixation was 2.22%, the incidence of early SSI was 1.24%, and the incidence of delayed SSI was 0.98%. If the main infection site of early infection is in the incision, debridement should be the main treatment method; if the infection site is around the internal fixation, implant replacement is recommended on the basis of debridement. Once delayed infection is diagnosed, implant removal is suggested.

## 1. Introduction

Surgical site infection (SSI) is a serious complication after lumbar spine surgery that increases the length of hospital stay, medical expenses, and rate of unplanned reoperations, bringing great challenges to both doctors and patients [1, 2]. At present, lumbar spine surgery that requires internal fixation is becoming increasingly common [3, 4]. Picada et al. [5] reported that the incidence of SSI in deep tissue after lumbar fusion and internal fixation was 3.2%. Reames et al. [6] reported that the incidence of SSI after pediatric scoliosis correction surgery was 2.6% (505/19360). Zhou et al. [7] conducted a meta-analysis including 603 cases of SSI in 22475 spine surgeries, with an incidence of 3.1%; the incidences in the cervical, thoracic, and lumbar spines were 3.4%, 3.7%, and 2.7%, respectively.

Lumbar transpedicular dynamic fixation could preserve the mobility of the fixed segment, maintain the height of the intervertebral space, and reduce adjacent segment degeneration [8, 9]. The Dynesys system is a representative transpedicular dynamic instrument that has been used clinically for more than 20 years [10, 11]. Correspondingly, lumbar Dynesys dynamic internal fixation also had a certain SSI incidence. For example, Welch et al. [12] and Grob et al. [13] reported that the infection rate after Dynesys dynamic stabilization was 0.9% (1/101) and 3.2% (1/31). However, their sample size was limited, and the infection rates were not representative.

At present, the application of lumbar Dynesys dynamic internal fixation is not widespread. The most published literature mainly reports its clinical efficacy and imaging changes [11, 14, 15]. To our knowledge, there are no studies that have specifically reported on postoperative infection following lumbar Dynesys dynamic internal fixation. Therefore, the author retrospectively researched the incidence of SSI following more than 1000 cases of lumbar Dynesys dynamic fixation and its postoperative management strategy.

## 2. Materials and Methods

A retrospective study of all cases after lumbar Dynesys dynamic internal fixation performed by the author's team from January 2010 to December 2019 was performed, and the data from patients with SSI were collected. This research project was reviewed and approved by the Scientific Research Ethics Committee of Southwest Hospital, Army (Third) Military Medical University.

### 2.1. Inclusion and Exclusion Criteria

Inclusion criteria were as follows: (1) cases diagnosed as lumbar degenerative disease and following lumbar Dynesys dynamic internal fixation; (2) followed up for more than 12 months; and (3) the main observational content must include management after SSI was diagnosed.

Exclusion criteria were as follows: (1) revision surgery and (2) if the clinical data were incomplete.

### 2.2. Diagnosis of SSI

The SSI after the surgery or during the follow-up could occur at the incision (skin and subcutaneous tissue) or below the deep fascia, spinal canal, intervertebral space, paravertebral space, and around the internal instrument [16, 17]. Diagnostic criteria: (1) clinical manifestations included fever, low back pain and/or lower limb radiating pain, swelling, exudation, sinus around the incision, etc.; (2) inflammatory indicators were increased, such as white blood cells (WBC), neutrophils (N), thrombocytes, C-reactive protein (CRP), erythrocyte sedimentation rate (ESR), and procalcitonin; (3) MRI showed that the low signal on the T1-weighted image and the high signal or mixed signal on T2-weighted image in subfascial tissue around the surgical site; (4) color ultrasound indicated that there was an abscess in the surgical site; and (5) bacterial culture was positive. (1) was the main criterion, (2), (3), (4), and (5) were the secondary criteria, and the SSI was diagnosed by meeting (1) and any one of (2)–(5).

### 2.3. Treatment Methods

#### 2.3.1. Antibiotic Treatment

Vancomycin and (or) imipenem and cilastatin sodium were early selected empirically, and subsequently sensitive antibiotics were selected based on pathogenic bacteria and drug susceptibility test results. The total course of treatment was 8–12 weeks, and intravenous medication was performed for 4∼8 weeks and then oral antibiotics for 4 weeks.

#### 2.3.2. Surgical Intervention

(1) The incision was healed without local infection, but MRI showed some localized fluid around the internal fixation with no high or slightly higher inflammatory indicators. Puncture aspiration could be used to retain specimens, and repeated puncture and irrigation was suggested. (2) If the infection was only confined to the skin and subcutaneous tissues without involving the internal fixation below the deep fascia, a thorough debridement and suture was recommended. (3) If the infection was around the spacer below the deep fascia, and the pedicle screw did not show bone absorption “halo” sign in X-ray, it was advisable to take out the spacer and connector, completely debride, and then install new spacers and connectors. (4) If the infection mainly occurred below the deep fascia, the internal instrument was soaked with pus, the pedicle screw showed bone absorption “halo” sign in X-ray, the pedicle screw was loosening during surgery, the infection involved the screw trajectory in the vertebral body, and/or paravertebral abscess/the psoas major muscle abscess was formed, removal of the internal instrument should be performed.

#### 2.3.3. Systemic Supportive Treatment

Albumin was supplemented for correcting hypoalbuminemia and anemia and maintaining albumin above 35 g/L and hemoglobin above 90 g/L.

### 2.4. Observational Indicators and Grouping

Incidence of SSI: the patients' age, sex, smoking and drinking behavior, previous surgical history, primary disease, and concomitant disease; intraoperative conditions: number of fenestrations, number of discectomy, number of fixed segments, operation time, blood loss, blood transfusion, and dural rupture; and postoperative infection time, symptoms, inflammatory indicators, pathogenic bacteria, and treatment. Infection that occurred within 3 months after lumbar Dynesys dynamic internal fixation was defined as an early infection, and infection that occurred 3 months after lumbar Dynesys dynamic internal fixation was defined as a delayed infection [18, 19]. The infected cases were divided into two groups, namely, the early infection group and delayed infection group, referred to as Groups A and B, respectively. The relevant indicators and main treatments of the two groups were compared.

### 2.5. Statistical Analysis

SPSS (version 19.0 IBM, NY, USA) software package was used for statistical analysis. Count data were recorded as yes or no, and measurement data were recorded as mean ± SD. For comparison between Groups A and B, the enumeration data were analyzed using the chi-square test or Fisher's exact test. If the measurement data were normally distributed, the independent-sample *t*-test was used; if the measurement data were not normally distributed, data conversion or Mann–Whitney test was used. *P* < 0.05 was taken to indicate that the difference was significant.

## 3. Results

A total of 1125 patients were followed up after lumbar Dynesys dynamic internal fixation, including 663 cases of lumbar disc herniation, 201 cases of lumbar spinal stenosis, 115 cases of lumbar spondylolisthesis, 71 cases of lumbar degenerative scoliosis, and 75 cases of lumbar discogenic pain. Twenty-five cases of SSI occurred, and the incidence of SSI was 2.22% (25/1125). There were 14 cases of early infection, with an infection rate of 1.24% (14/1125) and 11 cases of delayed infection, with an infection rate of 0.98% (11/1125).

### 3.1. General Information of the Patients and Surgical Details

The twenty-five patients included 21 males and 4 females, aged 49.4 ± 18.2 years (21–78). The primary diseases were lumbar disc herniation, lumbar spinal stenosis, lumbar degenerative spondylolisthesis, and lumbar degenerative scoliosis. There were 10 patients with a drinking history, 9 patients with a smoking history, 8 patients with hypertension, 4 patients with diabetes, and 4 patients with a history of lumbar surgery. The follow-up time was 58.4 ± 32.9 months (12–131). The number of fenestrations was 1.7 ± 1.0 (1–4), the number of discectomy was 1.2 ± 0.5 (0–2), and the number of fixed segments was 2.1 ± 0.811 (1–3). The operation time was 179 ± 74 minutes (80–330), the blood loss was 332 ± 253 ml (100–1200), and the blood transfusion was 154 ± 283 ml (0–1000). There was 1 case of dural rupture.

### 3.2. Postoperative Infection Time, Symptoms, and Inflammatory Indicators of SSI

Fourteen cases of early infection occurred 12.3 ± 8.3 days postoperatively (3–30); and 11 cases of delayed infection occurred 33.3 ± 18.9 months postoperatively (3–62). The main symptoms were low back pain (or lower limb radiating pain), incision exudation, redness and swelling, and fluid accumulation. Some inflammatory indicators increased, such as white blood cells, the percentage of neutrophils, platelets, C-reactive protein, erythrocyte sedimentation rate, and procalcitonin.

### 3.3. Secondary Surgery for SSI and Pathogenic Bacteria

A total of 20 cases underwent secondary surgery. The surgical methods mainly included debridement, implant replacement, and implant removal. The other 3 patients underwent puncture (irrigation), and 2 patients received only antibiotic treatment. Eleven cases of pathogenic bacteria were identified, accounting for 44%, 13 cases had negative cultures, and no specimens could be cultured in 1 case. Pathogenic bacteria included 4 cases of Staphylococcus epidermidis, 2 cases of Staphylococcus aureus, and 1 case each of Salmonella, Pseudomonas aeruginosa, Enterobacter, Acinetobacter baumannii, and Streptococcus lactis. The follow-up time after the second surgery for SSI was 42.2 ± 25.1 months (5–105), and there was no reinfection during the follow-up period.

### 3.4. Comparison of Surgical Details, Clinical Symptoms, Inflammatory Indicators, Pathogen Detection Rate, Main Infection Site, and Main Treatment Measures between Groups A and B (Table 1)

There were no significant differences in the number of fenestrations, the number of discectomys, the number of fixed segments, operation time, blood loss, or blood transfusions between Groups A and B (*P* > 0.05). The fixed segment, operation time, blood loss, and blood transfusion in Group A were slightly higher than those in Group B. The inflammatory indicators of Group A were significantly higher than those of Group B (all *P* < 0.05), except for procalcitonin. The detection rates of pathogenic bacteria in Groups A and B were 62.5% and 27.3%, respectively (*P* > 0.05). The main infection sites in Group A were located at the skin and subcutaneous tissue and around the internal instrument, while the main infection sites in Group B were located around the internal instrument. Group A mainly used treatment measures such as debridement, implant replacement, and mere antibiotics. Group B mainly used treatment measures such as implant removal and puncture (irrigation).

**Figure 1:**
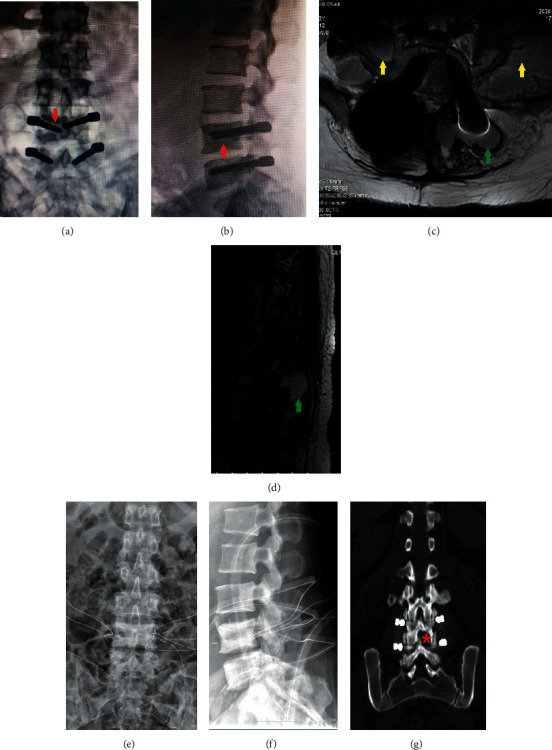
(a, b) Three years after L4-5 Dynesys dynamic internal fixation, anteroposterior and lateral X-view: the red arrow indicates bone resorption and loosening of the pedicle screw (halo sign). (c, d) Lumbar MRI: the green arrows indicate empyema around pedicle screws and spacers, and the yellow arrows indicate bilateral psoas muscle abscesses. (e, f) Placing drainage tubes after removal of the implant. (g) Opening a window between the lamina, retaining the lateral 1/2 of the inferior articular process in L4.

The typical case is shown in Figure 1. A 40-year-old female patient with low back pain and left lower limb pain for 4 days was admitted to the hospital on July 6, 2020. Three years prior, she had undergone L4-5 discectomy and Dynesys dynamic internal fixation due to L4-5 disc herniation. She had a history of diabetes for 3 years. Laboratory results showed WBC 18.9 × 10^9^/L, neutrophil 91.2%, CRP 170.0 mg/L↑, procalcitonin 0.35 ng/ml, and ESR 120 mm/h. Lumbar X and MRI results showed loose internal fixation, empyema around the internal fixation, and psoas major abscess (Figure 1). The diagnosis was delayed SSI after lumbar internal fixation. Treatment measures were implant removal, debridement, drainage, antibiotic therapy, support, and other treatments.

## 4. Discussion

In this research, 24 patients who underwent lumbar Dynesys internal fixation had SSI, with an infection rate of 2.22%. Goldstein et al. [20] reported 10 patients undergoing Dynesys dynamic surgery, of whom 3 cases had deep wound infections, with an infection rate as high as 30%. Pham et al. [21] reviewed the complications after Dynesys fixation. A total of 21 studies included 1166 patients, the average follow-up time was 33.7 months (12.0–81.6), and the incidence of SSI was 4.3%. Wiseman et al. [22] believed that titanium and titanium alloy compounds were less likely to be infected at the surgical site than other implant materials, including polymethylmethacrylate (PMMA), stainless steel, and hydroxyapatite. Titanium is one of the best implant materials compatible with human tissues, especially for fixing bones. The surface of titanium and titanium alloys was easily colonized by osteoblasts and soft tissues, thereby preventing the adhesion and colonization of bacteria and other pathogenic microorganisms on the surface of the internal instrument [23]. The pedicle screws of the Dynesys system are not connected by titanium rods but by a combination of a connector and spacer. The connector is woven from polyethylene terephthalate materials, and the spacer is made up of polycarbonate polyurethane. There is no soft tissue growth in the gap between the connector and the spacer, and the gap between the spacer and the pedicle screw during spinal flexion and extension activities might be where bacteria colonize. At the same time, the braided suture of the connector has greater bacterial adhesion, which might increase the likelihood of infection [24]. Goldstein et al. [20] postulated that intraoperative bacteria entered the surgical site, and the spacer acted as a medium for bacteria. However, the sample size was only 10 cases, and the results were hard to be convinced.

This study showed that the age, fixed segment, operation time, blood loss, and blood transfusion in Group A were higher than those in Group B, indicating that elderly patients and those with greater surgical trauma were prone to early SSI perioperatively. Early infection mainly manifested as incision exudation, low back pain (leg pain), and hydrops in the surgical site, while delayed infection mainly manifested as low back pain, sinus tract, abcess, etc. The inflammatory indicators were increased in most cases of early infection, while they were mostly normal in cases of delayed infection. The main infection site of early infection was located at the skin, subcutaneous tissue, and around the internal fixation, while the main infection site of delayed infection was located around the internal instrument. When delayed infection was suspected, MRI was performed. The hydrops around the internal instrument had obvious changes on the MRI, such as the high signal around the screw on the T2 image. The second invasive operation for early infection was mainly debridement, with complete removal of necrotic and inactivated tissue, and drainage and sealing of the incision. Early infection mainly occurred in the incision, and deep cavity infection was not common. If the infection around the internal instrument was serious, then replacement of the connector and spacer should be considered. The pedicle screw cannot be easily loosened in cases of early infection, so the screw might not need to be replaced. The author advocates the use of chlorhexidine (or iodophor), hydrogen peroxide, and physiological saline to repeatedly wash the infection site. For delayed infection, the main infection site was around the internal instrument, so for most patients, the internal instrument need to be removed.

Regardless of early infection or delayed infection, there are fewer concerns regarding lumbar dynamic stabilization surgery than lumbar fusion. Posterior (transforaminal) lumbar interbody fusion damages the most posterior spine structure, such as the lamina and facet joints. In early infection, implant removal would cause intervertebral instability, false joint formation, and increased neurological dysfunction. Lumbar dynamic stabilization does not require an intervertebral cage, avoiding the difficulty of removing intervertebral implants. The author has always advocated opening a window between the lamina, retaining the lateral 1/2 of the inferior articular process (Figure 1(g)) and achieving complete decompression of the nerve root canal by subtly expanding the lateral recess. Even if bilateral decompression is performed at the same level, the spinous process and the upper part of the bilateral lamina could be retained. Therefore, in patients undergoing lumbar dynamic stabilization, most of the posterior structure can be preserved, maintaining the stability of the spine. Once SSI occurs in lumbar dynamic internal fixation, implant removal has almost no effect on the stability of the spine. Of course, there is no “gold standard” for implant removal or retention in SSI after lumbar dynamic internal fixation, and it depends mainly on the unique situation of the patient, such as infection site, infection severity, patient's general condition, nutritional status, pathogenic bacteria, drug susceptibility test, treatment affordability, compliance, and other factors [25, 26].

**Figure 2:**
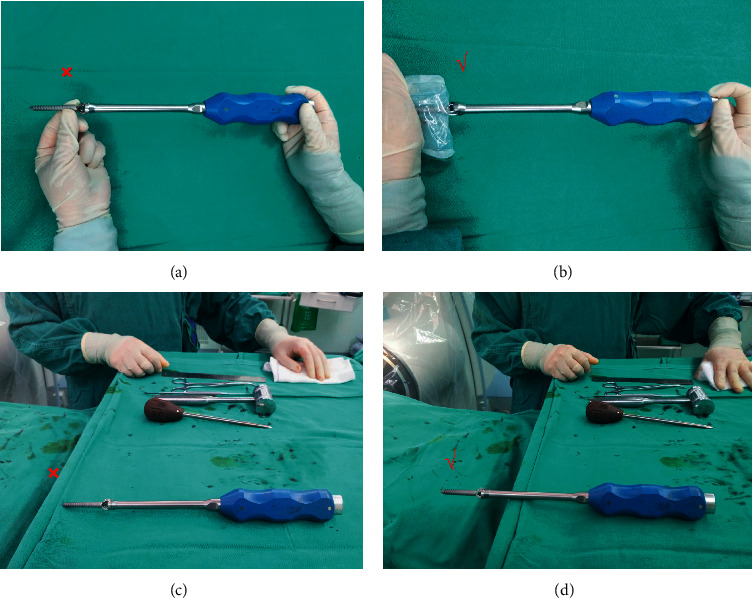
(a) Wrong: Dynesys screw was installed with direct touching; (b) correct: Dynesys screw was installed without direct touching. (c) Wrong: Dynesys screw was placed touching the cloth; (d) correct: Dynesys screw was placed without touching the cloth.

In terms of how to prevent and treat SSI after lumbar dynamic internal fixation, the author has some suggestions. Full attention should be paid to the risk factors for infection. Janssen et al. [27] pointed out that age, body mass index, American Society of Anesthesiologists (ASA) score, revision surgery, and the use of nonsteroidal anti-inflammatory drugs are risk factors for SSI after thoracolumbar internal fixation in adults. Other studies have indicated that a modified Glasgow prognostic score ≧1, BMI ≦20.39 kg/m^2^ [28], postoperative hyperglycemia, poor postoperative blood glucose control [29], perioperative hypoalbuminemia, and chronic steroid use are risk factors for SSI in spinal internal fixation [30]. The use of prophylactic antibiotics during the perioperative period and the correction of anemia and hypoalbuminemia are very important [31, 32]. A strict aseptic technique should be the basis, and direct contact with the internal instrument should be avoided as much as possible (Figure 2). For example, the Dynesys screw should be installed on the screwdriver without direct touching. In the screw implantation process, contact with gloves, cloth sheets, hooks, and muscle tissue should be avoided to the greatest extent possible. To ensure a sufficient extraversion angle of the screw and reduce the influence on the zygapophyseal joint, the author generally chose the Wiltse approach to complete the installation of the Dynesys system [33]. The muscle tissue should not be entrapped between the spacer and the screw because necrosis of the entrapped muscle is a good culture medium for bacteria. After the operation, the healing of the incision and inflammatory indicators needed to be carefully observed. Once SSI is suspected, specimens should be collected as soon as possible through incision exudate, drainage fluid, puncture fluid, etc. for pathogenic examination while using norvancomycin for empirical anti-infective therapy. After the drug sensitivity test is returned, the antibiotics may need to be adjusted, with an anti-infectious treatment of 8 to 12 weeks. Tsubouchi et al. [25] believed that timely use of effective antibiotics could help preserve implants. Lener et al. [34] reported that sensitive antibiotics should be administered intravenously for 2–4 weeks or until CRP drops significantly, followed by oral antibiotics for 6–12 weeks. Petilon et al. [35] advocated intravenous antibiotics for ≥6 weeks, followed by oral antibiotics for several weeks. Kowalski et al. [36] noted that even if the pathogenic test result is negative, long-term antibiotics are more effective in controlling and eradicating infection than short-term antibiotics (80%:33%). Of course, antibiotics could never replace surgical treatments such as debridement, implant replacement, or removal.

## 5. Conclusion

The incidence of SSI following lumbar Dynesys dynamic internal fixation was 2.22%, the incidence of early infection was 1.24%, and the incidence of delayed infection was 0.98%. If the main infection site of early infection is in the incision, debridement should be the main treatment method; if the infection site is around the internal fixation, implant replacement is recommended on the basis of debridement. Once delayed infection is diagnosed, implant removal is suggested.

## Figures and Tables

**Table 1:** Comparison of the observational indicators between Groups A and B.

		Group A	Group B	*P*
Age (years)		56.9 ± 18.4	39.8 ± 13.0	0.016

Surgical situation	Number of fenestration	1.7 ± 1.1	1.7 ± 1.0	0.903^**∗**^
	Number of discectomy	1.1 ± 0.4	1.2 ± 0.6	0.769^**∗**^
	Number of fixed segment	2.2 ± 0.9	1.9 ± 0.7	0.323^**∗**^
	Operation time (min)	195.8 ± 81.984	158.1 ± 70.7	0.208^**∗**^
	Blood loss (ml)	385.7 ± 293.2	263.6 ± 180.4	0.134^**∗**^
	Blood transfusion (ml)	182.1 ± 334.9	119.1 ± 210.8	0.809^**∗**^

Clinical symptom	Incision exudation	8	0	
	Low back pain (leg pain)	4	7	
	Incision hydrops	3	0	
	Red and swollen incision	3	1	0.007^#^
	Sinus tract	2	2	
	Fever	2	1	
	Abscess	0	3	

Inflammation indicator	WBC (×10^9^/L)	11.4 ± 3.0	8.2 ± 2.5	0.008
	*N* (%)	79.6 ± 12.3	68.9 ± 9.9	0.029
	Thrombocyte (×10^9^/L)	296.7 ± 88.4	222.8 ± 54.1	0.023
	SR (mm/1 h)	54.2 ± 26.8	34.2 ± 27.1	0.048^**∗**^
	CRP (mg/L)	64.9 ± 88.0	13.7 ± 15.8	0.012^**∗**^
	Procalcitonin (ng/ml)	2.5 ± 5.3	0.5 ± 1.0	0.639^**∗**^

Pathogenic bacteria	Positive rate	8/14 (57.1%)	3/11 (27.2%)	0.467^#^

Main infection site	Skin and subcutaneous tissue	9	0	0.001^#^
	Around internal instrument	3	10	
	Spinal canal	1	1	
	Intervertebral space	1	0	

Main treatment	Mere antibiotics	2	0	0.001^#^
	Puncture (irrigation)	1	2	
	Debridement	8	1	
	Implant replacement	2	0	
	Implant removal	1	8	

^∗^Mann-Whitney test; ^#^Fisher's exact test.

## Data Availability

The underlying data supporting the results of our study can be obtained by contacting the corresponding author via zhouqiang@hospital.cqmu.edu.cn.
